# Supplementary material for: Transcriptome of Left Ventricle and Sinoatrial Node in Young and Old C57 Mice
Source: Fortune J Health Sci. Author manuscript; Available in PMC 2023 Nov 2. (PMC10621664; doi:10.26502/fjhs.134)
Supplement: 1 [file NIHMS1934321-supplement-1.pdf]

Supplementary Figure

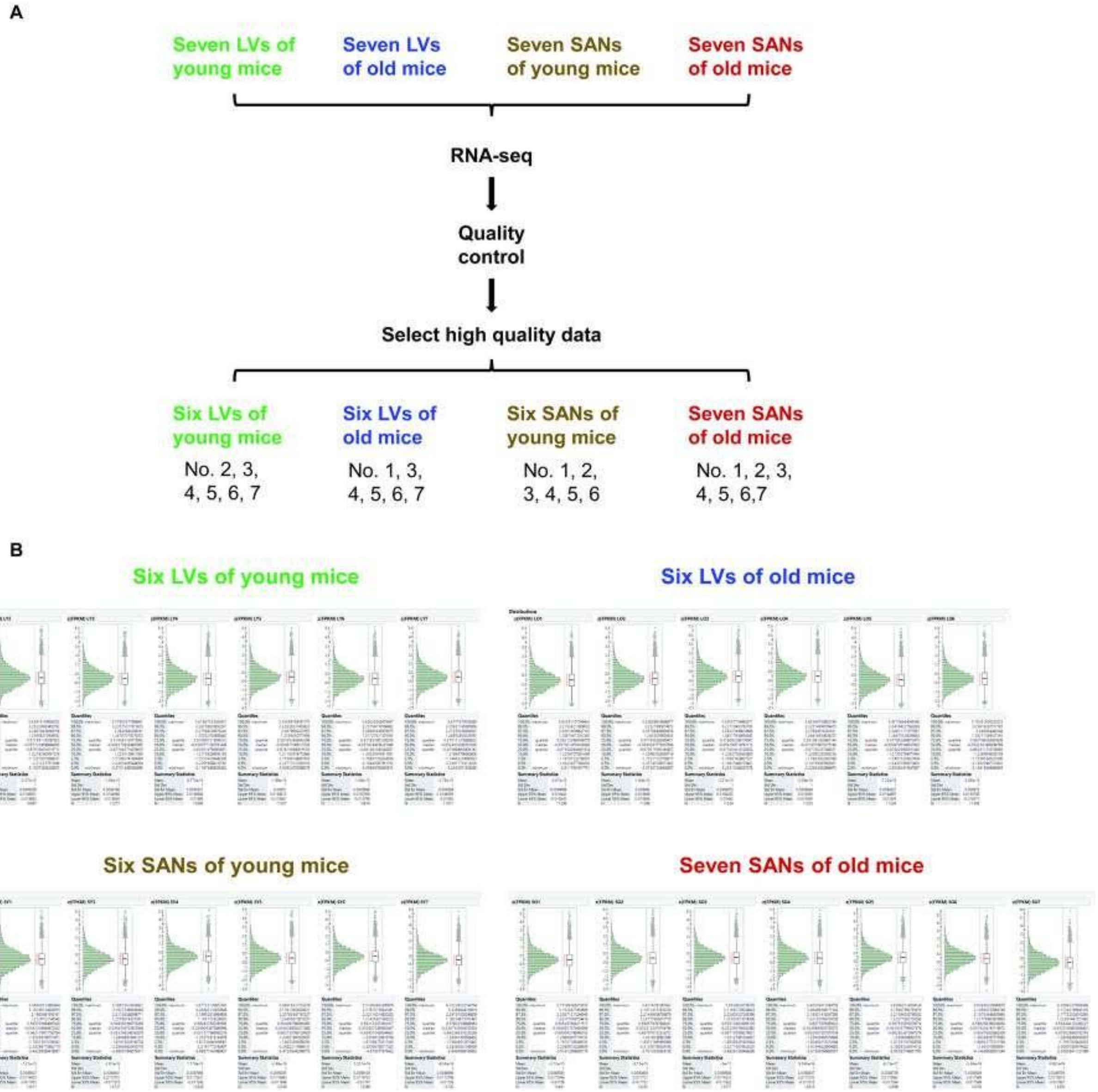

Supplementary Figure 1. Four groups of samples and the distribution of their FPKM values. A. Four groups of samples were included in this project: left ventricles (LVs) of young mice (LY), LVs of old mice (LO), sinoatrial nodes (SANs) of young mice (SY), and SANs of old mice (SO). After quality control, six samples in the LY group, six samples in the LO group, six samples in the SY group, and seven samples in the SO group were remained for subsequent analyses. **B.** The FPKM of each sample was z-score normalized to be in the approximate normal distribution.

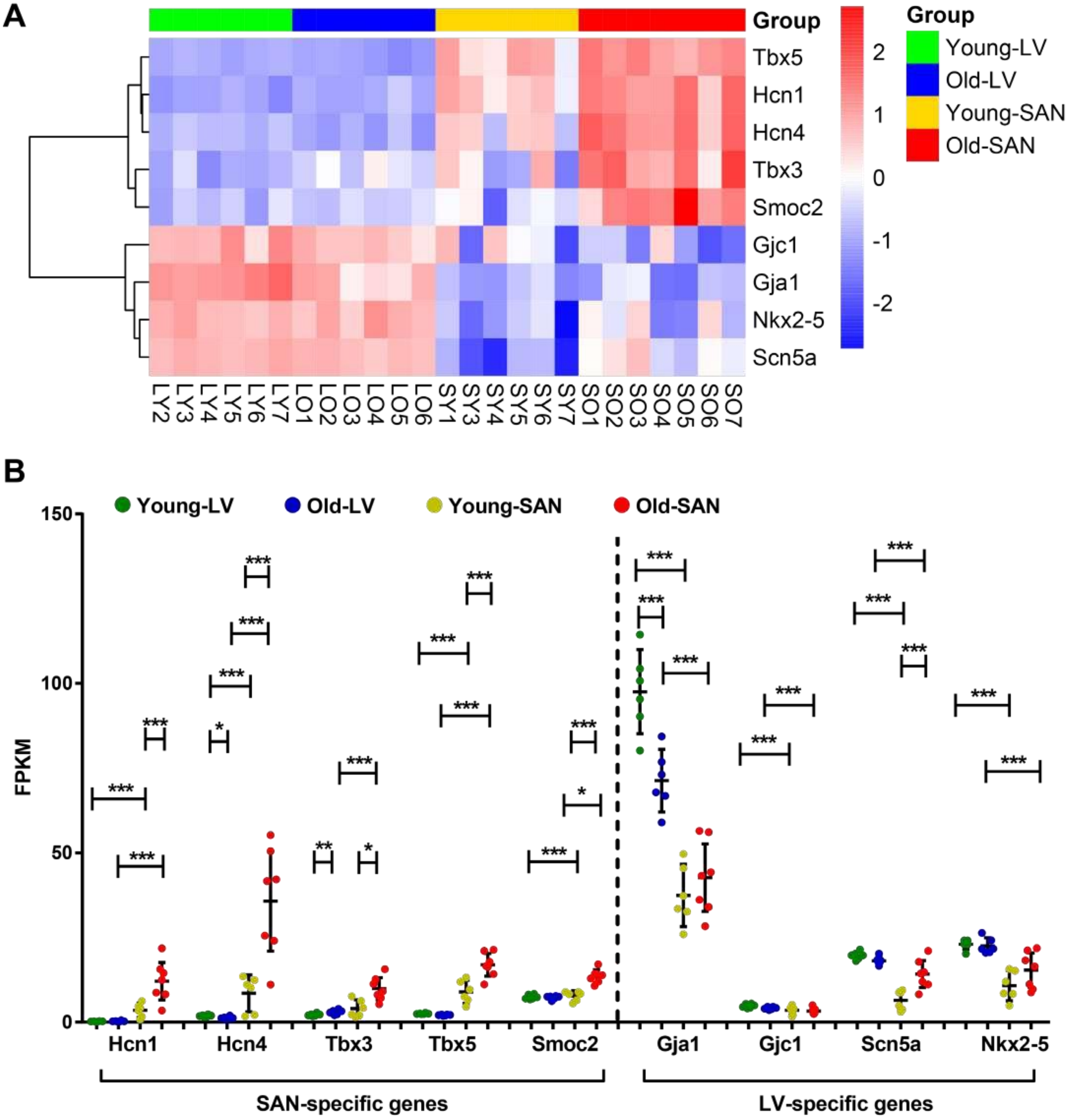

Supplementary Figure 2: Heart tissue marker genes. A. Heatmap of several heart tissue marker genes. B. Scatter plot of those heart tissue marker genes with the q-values between two groups: *, q-value < 0.05; **, q-value < 0.01; ***, q-value < 0.001. The bar shows the mean and 95% CI.

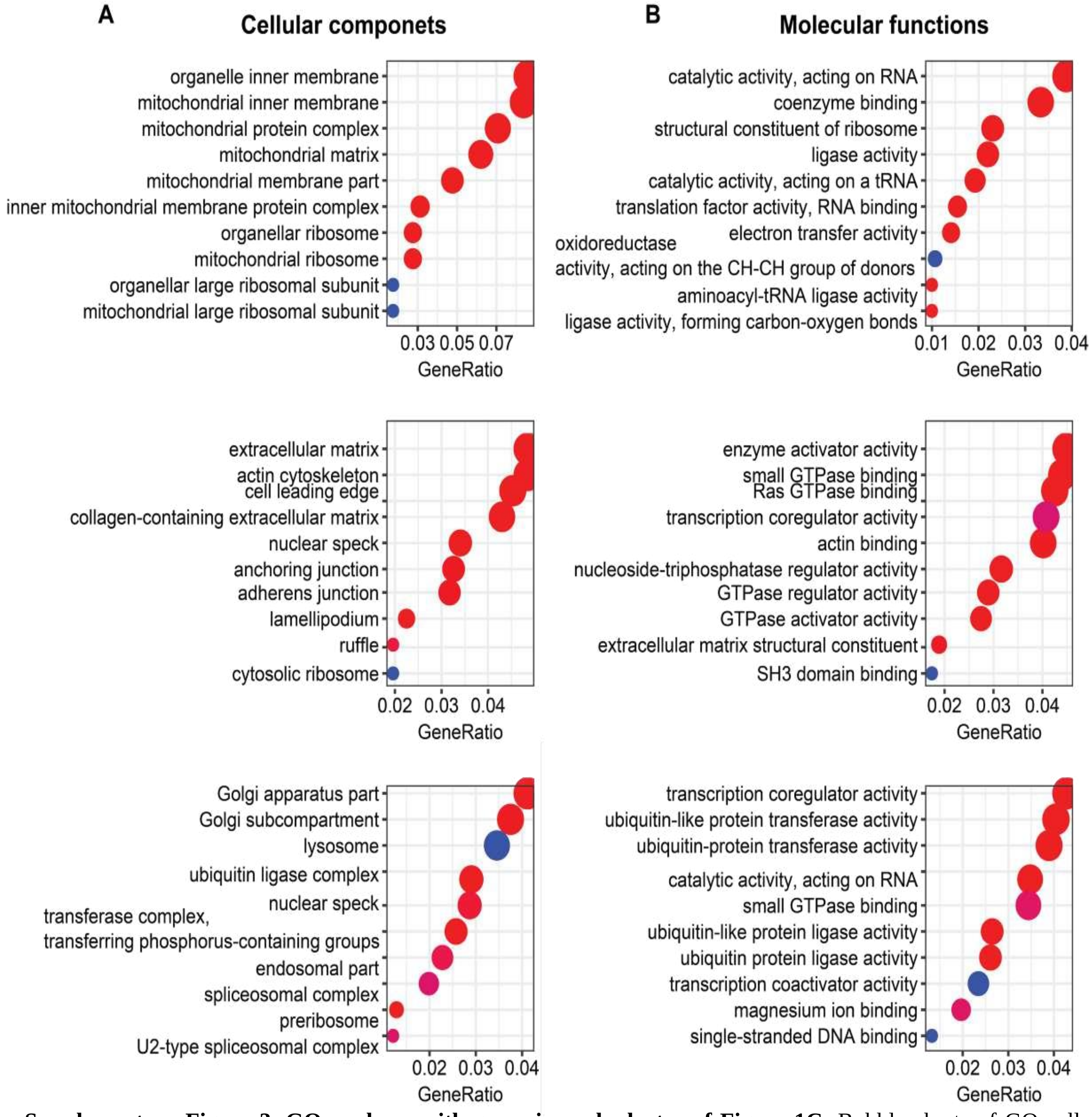

Supplementary Figure 3. GO analyses with genes in each cluster of Figure 1C. Bubble charts of GO cellular component (**A**) and molecular function (**B**) analysis with genes in each cluster. Same to Figure 1D and 1E, the larger the bubble, the more identified genes in the term. The redder, the less adjusted p-value, indicating more significant.

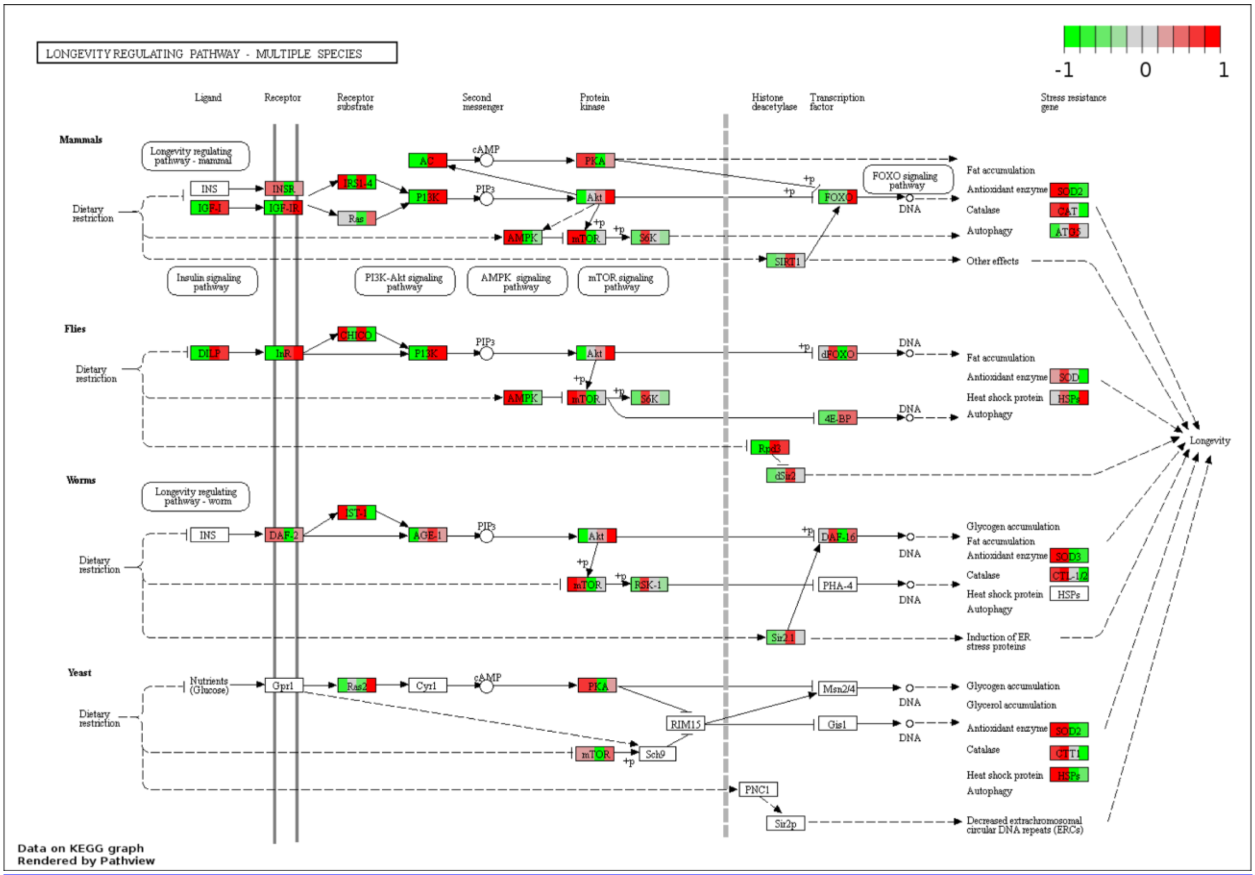

Supplementary Figure 4. Visualization of the entire longevity regulating pathway (multiple species) whose genes were clustered in the downmost cluster of Figure 1C. The longevity regulating pathway was visualized and compared in multiple species, including mammals, flies, worms and yeast.

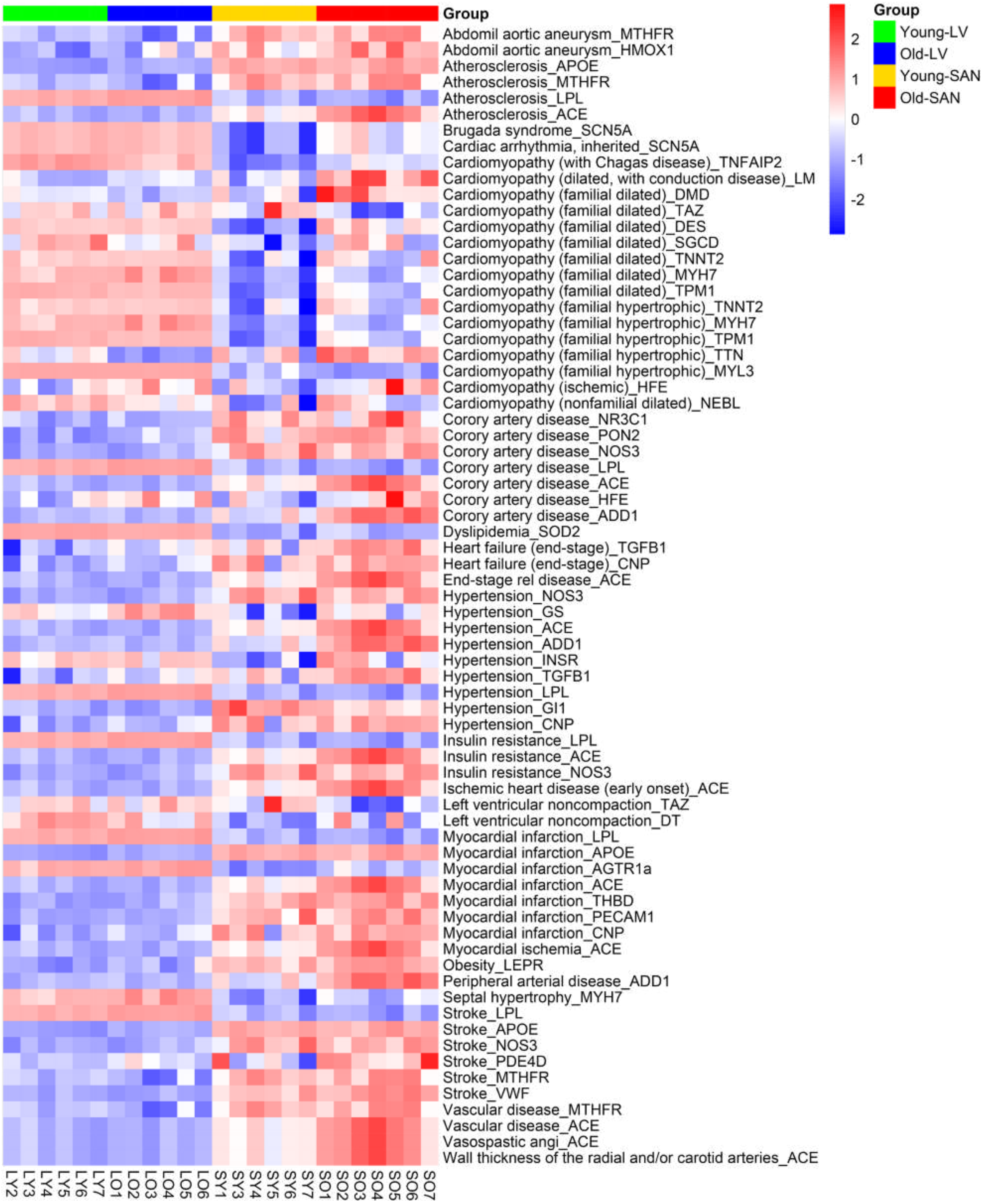

Supplementary Figure 5: Cardiovascular disease marker genes analysis. Heatmap of cardiovascular disease marker genes.

Young (SAN/LV)

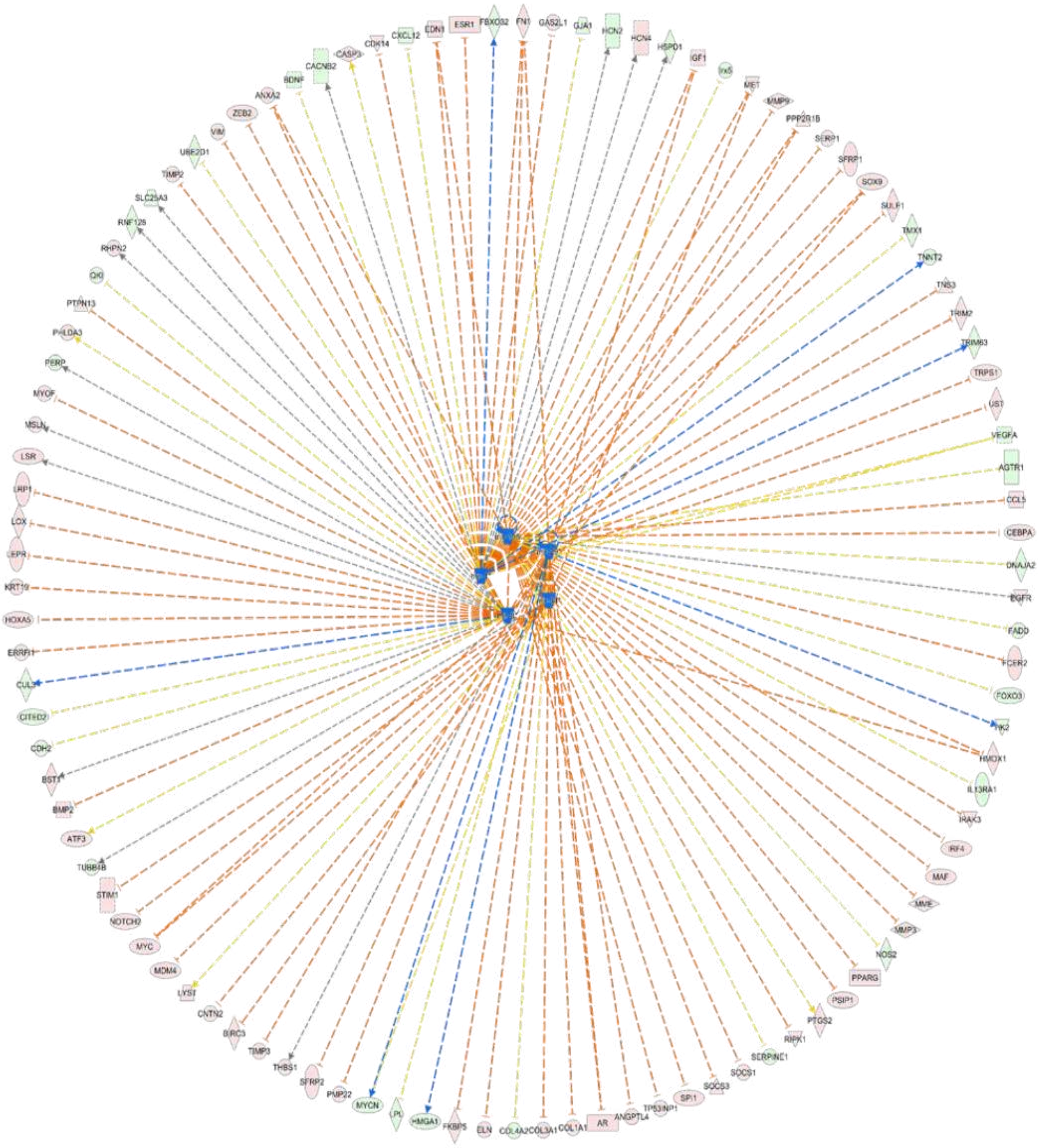

Supplementary Figure 6. Regulation of miRNAs on differentially expressed genes between SAN and LV tissues in the young mice. Genes identified in the RNA-seq are distributed in the circle, and predicted upstream mature miRNAs, mir-1, mir-155, mir-29, mir-34 and mir-155, are in the center.

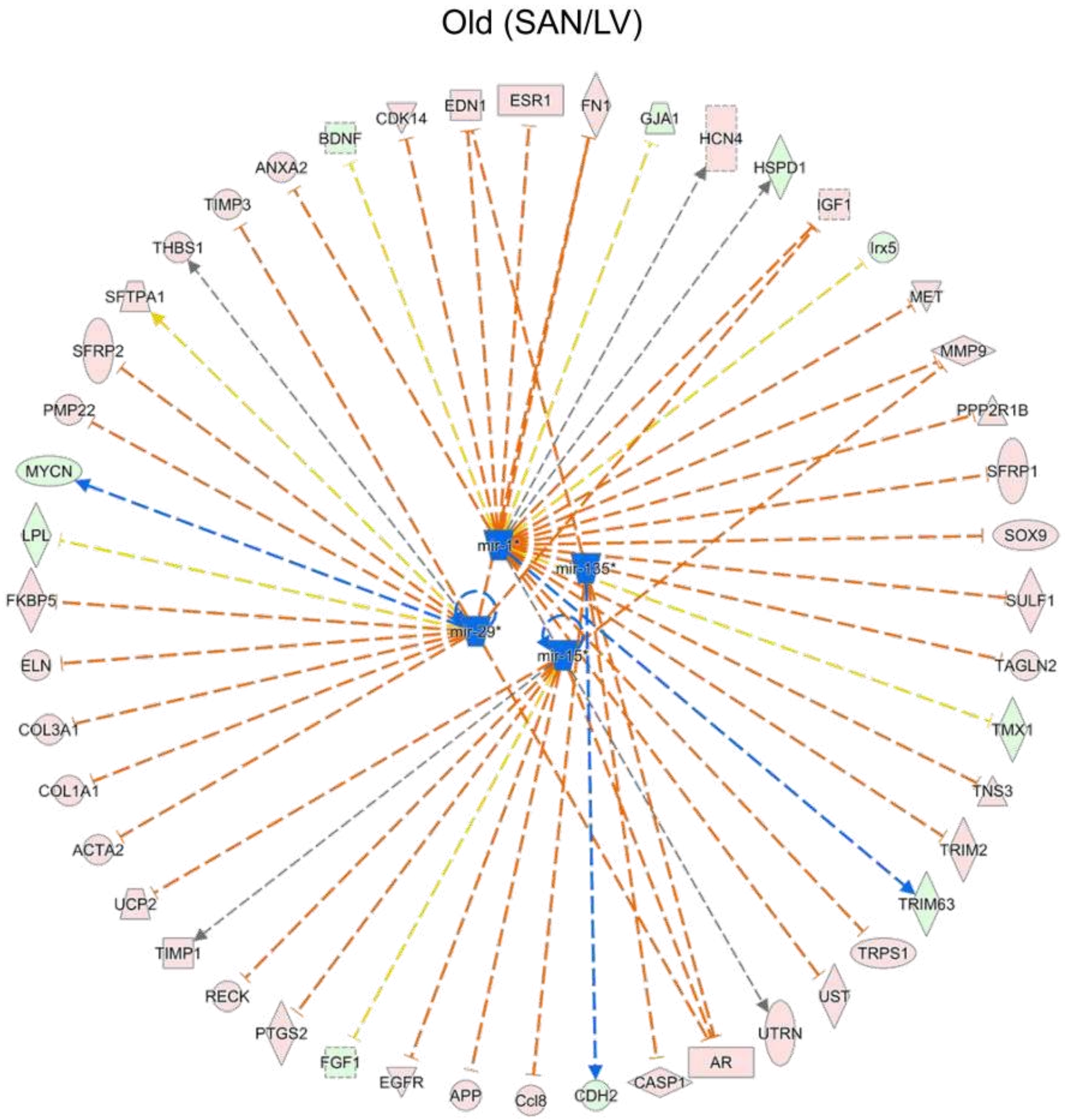

Supplementary Figure 7: Regulation of miRNAs on differentially expressed genes between SAN and LV tissues in the old mice. Genes identified in the RNA-seq are distributed in the circle, and predicted upstream mature miRNAs, mir-1, mir-135 and mir- 15, mir-29, are in the center.
